# Metagenomic Sequencing of an *In Vitro*-Simulated Microbial Community

**DOI:** 10.1371/journal.pone.0010209

**Published:** 2010-04-16

**Authors:** Jenna L. Morgan, Aaron E. Darling, Jonathan A. Eisen

**Affiliations:** 1 Department of Medical Microbiology and Immunology, University of California Davis, Davis, California, United States of America; 2 Department of Evolution and Ecology, University of California Davis, Davis, California, United States of America; 3 United States Department of Energy Joint Genome Institute, Walnut Creek, California, United States of America; Universidad Miguel Hernandez, Spain

## Abstract

**Background:**

Microbial life dominates the earth, but many species are difficult or even impossible to study under laboratory conditions. Sequencing DNA directly from the environment, a technique commonly referred to as metagenomics, is an important tool for cataloging microbial life. This culture-independent approach involves collecting samples that include microbes in them, extracting DNA from the samples, and sequencing the DNA. A sample may contain many different microorganisms, macroorganisms, and even free-floating environmental DNA. A fundamental challenge in metagenomics has been estimating the abundance of organisms in a sample based on the frequency with which the organism's DNA was observed in reads generated via DNA sequencing.

**Methodology/Principal Findings:**

We created mixtures of ten microbial species for which genome sequences are known. Each mixture contained an equal number of cells of each species. We then extracted DNA from the mixtures, sequenced the DNA, and measured the frequency with which genomic regions from each organism was observed in the sequenced DNA. We found that the observed frequency of reads mapping to each organism did not reflect the equal numbers of cells that were known to be included in each mixture. The relative organism abundances varied significantly depending on the DNA extraction and sequencing protocol utilized.

**Conclusions/Significance:**

We describe a new data resource for measuring the accuracy of metagenomic binning methods, created by *in vitro*-simulation of a metagenomic community. Our *in vitro* simulation can be used to complement previous *in silico* benchmark studies. In constructing a synthetic community and sequencing its metagenome, we encountered several sources of observation bias that likely affect most metagenomic experiments to date and present challenges for comparative metagenomic studies. DNA preparation methods have a particularly profound effect in our study, implying that samples prepared with different protocols are not suitable for comparative metagenomics.

## Introduction

The vast majority of life on earth is microbial, and efforts to study many of these organisms via laboratory culture have met with limited success, leading to use of the term “the uncultured majority” when describing microbial life on earth [1]. Metagenomics holds promise as a means to access the uncultured majority [2], [3], and can be broadly defined as the study of microbial communities using high-throughput DNA sequencing technology without requirement for laboratory culture [4]–[7]. Metagenomics might also offer insights into population dynamics of microbial communities [8], [9] and the roles played by individual community members [10]. Toward that end, a typical metagenomic sequencing experiment will identify a community of interest, isolate total genomic DNA from that community, and perform high throughput sequencing of random DNA fragments in the isolated DNA. The procedure is commonly referred to as shotgun metagenomics or environmental shotgun sequencing. Sequence reads can then be assembled in the case of a low-complexity sample [10], or assigned to taxonomic groupings using various binning strategies without prior assembly [5], [7], [11]. As binning is a difficult problem, many methods have been developed, each with their own strengths [11]–[17].

Assuming the shotgun metagenomics protocol represents an unbiased sampling of the community, one could analyze such data to infer the abundance of individual species or functional units such as genes across different communities and through time. However, many sources of bias may exist in a shotgun metagenomics protocol. These biases are not unique to random sequencing of environmental DNA. They have also been addressed in studies of uncultured microbial communities using PCR-amplified 16S rRNA sequence data. For example, it has been shown that differences in the cell wall and membrane structures may cause DNA extraction to be more or less effective from some organisms [18], [19], and differences in DNA sequencing protocol might introduce biases in the resulting sequences [20]. We also expect that methods to assign metagenomic reads to taxonomic groupings may introduce their own biases and performance limitations [16].

In selecting a particular metagenomic protocol, an awareness of alternative approaches and their limitations is essential. Towards this end, others have endeavored to benchmark the various steps of a typical metagenomic analysis. A few studies have attempted to quantify the efficiency and organismal bias of various DNA extraction protocols using environmental samples, but these have included unknown, indigenous microbes [18], [21]–[23]. One other benchmark of metagenomic protocols focused mainly on the informatic challenge of assigning reads from *a priori* unknown organisms to taxonomic groups in a reference phylogeny [16]. In that *in silico* simulation, the authors randomly sampled sequence reads from 113 isolate genomes, and mixed them to create three “communities” of varying complexity. While that type of informatic simulation of metagenomic reads is a useful approach for benchmarking different binning methods, the models used for such simulations simply can not capture all factors affecting read sampling from a real metagenome sequencing experiment. Even if the model complexity were increased, appropriate values would need to be experimentally determined for the new simulation model parameters.

In this work, we describe an *in vitro* metagenomic simulation intended to inform and complement the *in silico* simulations used by others for benchmarking. Using organisms for which completed genome sequences were available, we created mixtures of cells with equal quantities of each organism. We then isolated DNA from the mixtures and used two approaches to obtain sequence data. For all simulated metagenomic samples, we created small-insert clone libraries that were end-sequenced using Sanger chain termination sequencing [24] and capillary gel electrophoresis. For one of the samples, we generated additional sequence using the cloning-independent pyrosequencing method [25] on the Roche GS20. The resulting sequence data were then analyzed for biases introduced during metagenome sequencing. For this study, organisms were chosen to represent a breadth of phylogenetic distance, cell morphology, and genome characteristics in order to provide useful test data for benchmarking binning methods. This experiment was not designed to test specific hypotheses about how those factors or others may influence the distribution of reads in a metagenomic survey. Nevertheless, these data can be used to determine appropriate parameter ranges for metagenomic simulation studies, or directly as a test dataset for binning.

## Results and Discussion

### Constructing a simulated metagenome

**Figure 1:**
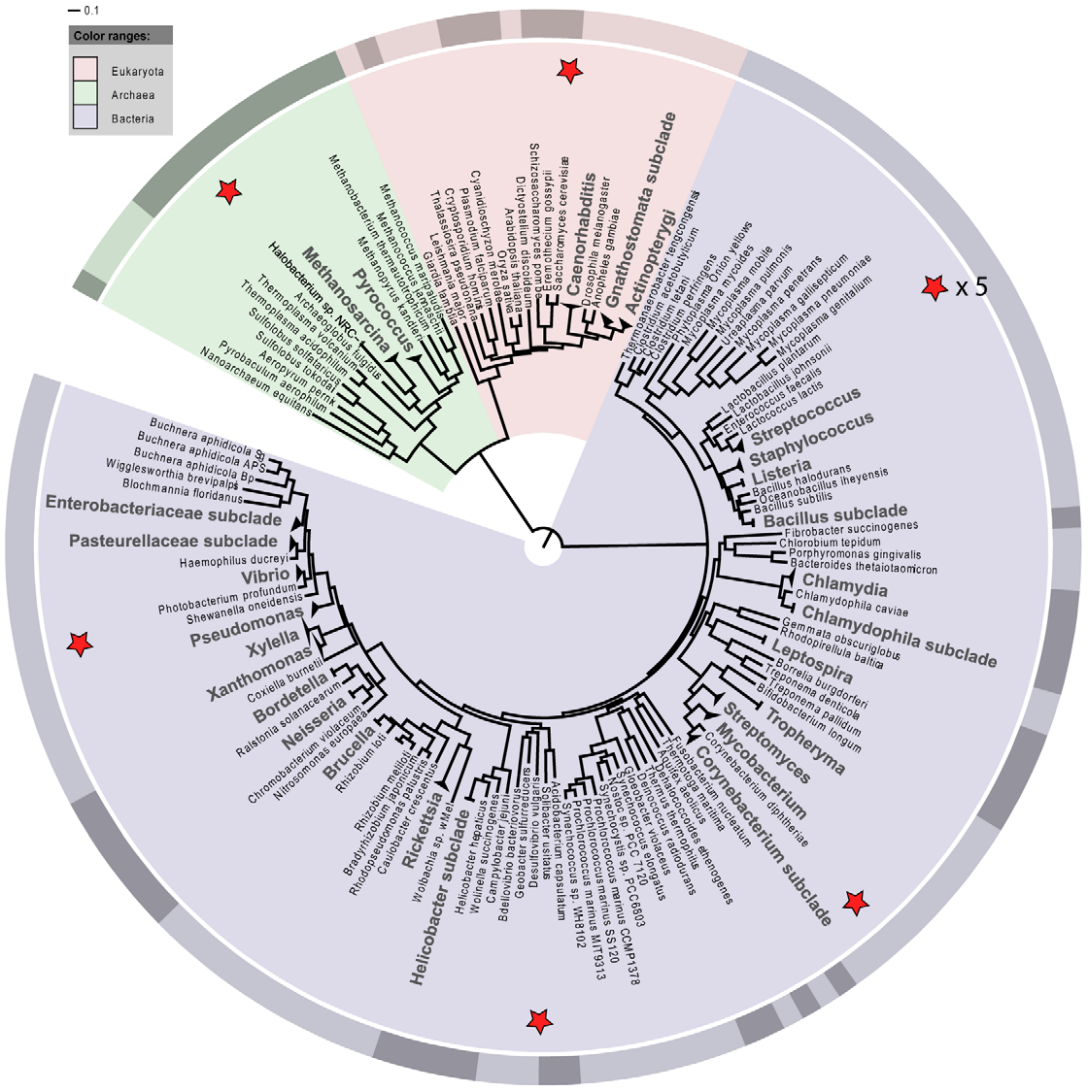
Phylogenetic distribution of organisms selected for the metagenomic simulation. A phylogenetic tree of three domains with representative groups is shown. Organisms used in this study are indicated by *. The organisms used represent all known domains of life, include four bacterial phyla, a variety of genome sizes, GC compositions, and cell wall types. Large font size indicates clades where multiple isolate genomes have been collapsed into a single leaf node.

Organism selection was guided by the data available in the Genomes On Line Database as of November 2007 [26]. Pathogens, obligate symbionts, and obligate anaerobes were removed from consideration for the simulated metagenome because these organisms are difficult to culture in our laboratory setting. We selected ten organisms representing all three domains of life and several levels of phylogenetic divergence. *Halobacterium sp*. NRC-1 [27] and *Saccharomyces cerevisiae* S288C [28] were selected to represent the archaeal and eukaryotic domains, respectively. Because it has been shown that cell membrane structure can have a significant effect on DNA extraction efficiency [18], [19], [22], we included both Gram-positive and Gram-negative bacterial species. Five relatively closely-related organisms were selected from among the lactic acid bacteria, a clade of low-GC, Gram-positive Firmicutes (*Pediococcus pentosaceous, Lactobacillus brevis, Lactobacillus casei, Lactococcus lactis cremoris* SK11, and *Lactococcus lactis cremoris* IL140) [29]. To provide phylogenetic breadth within the Bacteria, we also included *Myxococcus xanthus* DK 1622 [30] (a delta-proteobacterium), *Shewanella amazonensis* SB2B, GenBank Accession #CP000507, [31] (a gamma-proteobacterium), and *Acidothermus cellulolyticus* 11B [32] (an Actinobacterium). Figure 1 gives the placement of the organisms on the tree of life and Table 1 lists some general features of each organism. These ten organisms were not selected to represent a real, functional community, rather they were chosen to provide sequence data that would best allow the testing of the accuracy and specificity of various binning methods. To this end, we have chosen five phylogenetically diverse species with very different genome compositions and five species that are relatively closely related to each other, with very similar genome compositions.

**Table 1:** Characteristics of organisms in the simulated metagenome.

Taxonomic group	Organism	Genome size w/plasmids (Mb)	%GC	Cell density of stock culture	Culture volume into mixture (µL)	Concentration of isolate DNA extractions (ng/µl)	% reads expected in metagenomic reads
Lactic Acid Bacteria (Firmicutes)	*Lactococcus lacti*s cremoris IL1403	2529	35.7	175464	1096	141.09	7.75
Lactic Acid Bacteria (Firmicutes)	*Lactococcus lactis* cremoris SK11	2707	35.9	102564	1875	119.1	11.19
Lactic Acid Bacteria (Firmicutes)	*Pediococcus pentosaceous*	1800	37.4	1600000	120.19	414.41	2.5
Lactic Acid Bacteria (Firmicutes)	*Lactobacillus casei*	2988	46.6	909090	211.54	635.78	6.74
Lactic Acid Bacteria (Firmicutes)	*Lactobacillus brevis*	2398	46	333333	576.92	303.08	8.76
Gamma-proteobacteria	*Shewanella amazonensis* SB2B	4306	53	1025641	187.5	463.46	4.35
Delta-proteobacteria	*Myxococcus xanthus* DK	9139	68.9	200000	961.54	198.14	9.5
Actinobacteria	*Acidothermus cellulolyticus* 11B	2445	66.9	96154	2000	142.64	14.3
Eukaryota	*Saccharomyces cerevisiae* S288C	12096	38	196078	980.77	633.45	31.13
Archaea	*Halobacrerium sp.* NRC-1	2571	65.9	1000000	192.31	387.84	3.74

For each organism in the mixture, we give genome and organism characteristics alongside statistics for various stages of sample preparation. The cell density was determined by flow cytometry. The stock cultures were mixed such that each organism contributed an equal number of cells to the mixture. DNA from an aliquot of each culture was extracted using the EnzBB method and quantified. That quantity is used to calculate an expected representation of the organisms as a percentage of sequence reads, given in the column “% expected in metagenomic reads.”

**Figure 2:**
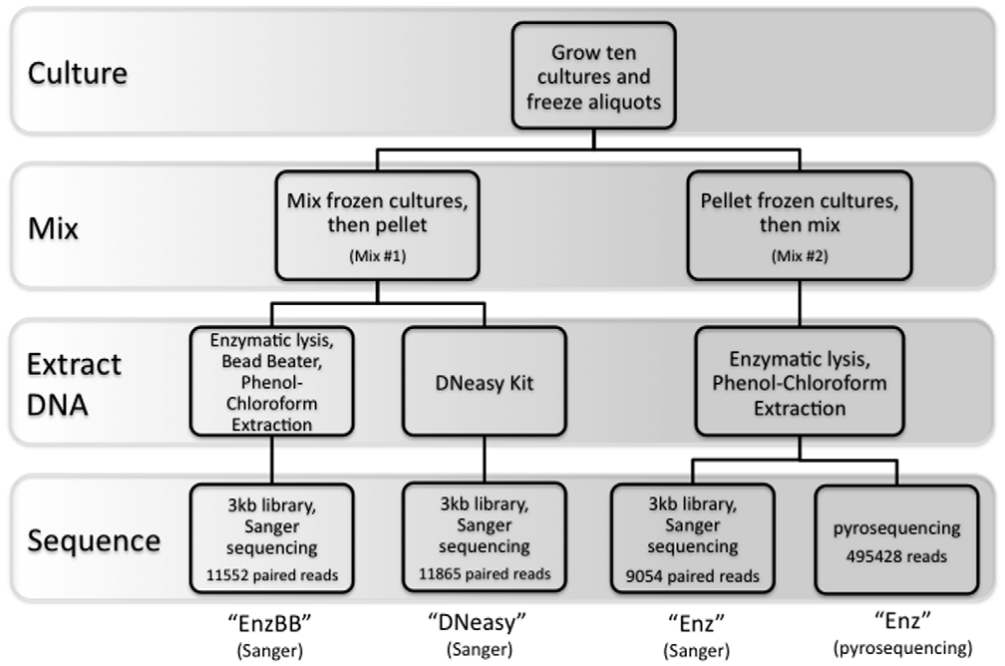
Outline of the steps involved in the creation and sequencing of the simulated metagenomic samples.

As described in Methods below, cultures for each organism were grown and cells from each culture were counted using flow cytometry. We then constructed two distinct simulated microbial communities that were made by mixing all organisms with different approaches (see Figure 2). The first approach involved mixing the cultures directly prior to extracting DNA from the collection of mixed cells. To this mixture, two DNA extraction techniques were applied in parallel, including an enzymatic extraction with a bead beater (referred to throughout as “EnzBB”), and the Qiagen DNeasy kit (referred to throughout as “DNeasy”). Preliminary sequence data from this mixture included no reads from the halophilic archaeon, *Halobacterium sp*. NRC-1. One possible explanation for this observation is that upon mixing, the high-salt culture medium in which the *Halobacterium* cells were growing was diluted, causing them to lyse. If cell lysis occured rapidly, before recovery of the mixed cell pellet, no DNA would be recovered from the lysed cells. To address this possibility, we made a second mixture of cells using a different approach. The second approach involved pelleting a known number of cells from each individual culture, mixing cell pellets, then performing DNA extraction on the mixed pellets using an enzymatic DNA extraction (referred to throughout as “Enz”). Simulated metagenomic DNA samples were then subjected to high-throughput sequencing using Sanger sequencing and pyrosequencing technologies (see Methods for a description of the sequencing protocols). Finally, to assess DNA extraction efficiency for each organism in isolation, an enzymatic extraction with a bead beating step (EnzBB) was applied to each isolate culture separately. Table 1 documents the quantification of total DNA extracted from each organism individually.

### Taxonomic Assignment of Reads

For each simulated metagenome, we used a BLAST search [33] to map quality-controlled reads back to the set of reference genomes, yielding a count of reads assigned to each organism (see Methods for details). A complete set of read mappings and summaries of the numbers of reads assigned to each organism is given in Table 2.

**Table 2:** Mapped reads.

	DNeasy			EnzBB			Enz			
	main	add	glyc	main	add	glyc	main	add	glyc	pyrosequencing
*Acidothermus*	64.21	60.57	48.96	28.53	46.34	34.96	16.08	47.97	43.43	13.66
*Halobacterium*	0.15	0.10	0.95	0.03	0.32	0.70	1.28	0.19	4.50	1.19
*Lb. brevis*	3.92	7.56	13.00	7.58	9.78	19.22	15.81	6.48	18.76	17.34
*Lb. casei*	4.21	4.88	1.99	9.23	6.60	4.46	13.97	5.98	1.57	15.41
*Lc. lactis* SK11	0.02	0.00	0.00	0.74	0.24	0.28	0.13	0.00	0.12	0.22
*Lc. lactis* Il1403	4.43	3.03	1.71	29.34	7.63	8.50	5.10	4.28	2.35	4.42
*Myxococcus*	7.99	7.71	4.46	4.18	2.78	5.29	33.19	5.06	11.32	27.58
*Pediococcus*	0.24	0.21	0.38	5.21	5.88	3.90	0.29	0.36	0.09	0.27
*Saccharomyces*	2.76	0.46	0.00	8.42	0.00	0.00	0.78	0.04	0.00	0.42
*Shewanella*	12.08	15.48	28.56	6.73	20.43	22.70	13.36	29.64	17.87	19.48
**Total mapped**	**11865**	**1945**	**1054**	**11552**	**1258**	**718**	**9054**	**5351**	**4338**	**495428**
**Total reads**	**14692**	**2625**	**1726**	**14418**	**2040**	**1348**	**11781**	**6542**	**6186**	**505962**

For each simulated metagenome (columns), the total number of reads is given at bottom and the proportion of reads mapped to each of the ten reference organisms are in rows. *Lb*  =  *Lactobacillus*, *Lc*  =  *Lactococcus*. For each of the three DNA extraction protocols, data are provided for the primary sequence libraries (“main”) as well as for the additional libraries, created using the same protocol with (“glyc”) and without (“add”) glycerol added to the stock isolate cultures.

Many reads did not map back to reference genomes using our stringent criteria. Such reads may represent highly conserved sequences that hit multiple genomes making unambiguous mapping impossible, had too few high-quality bases, or they may represent an unknown source of sequence library contamination. To further investigate the origins of unmapped reads, we searched those reads using BLAST against the NCBI non-redundant nucleotide database (see Table 3). We find that many unmapped reads do hit organisms present in our sample, but do so with less than 95% sequence identity. Sequencing errors, either in our data or in the published genome data, may contribute to this category of reads. In general, the lower identity reads follow the taxonomic abundance distribution of mapped high-identity reads. We also found a substantial number of hits to parts of a *Lactococcus* bacteriophage phismq8. This phage genome was not present (lysogenized) in either of the two reference *Lactococcus* genome sequences. All of the *Lactococcus* strains used for this study are the same strains, from the same lab, that were the source for the genome sequencing projects, suggesting that at least one of the *Lactococcus* cultures had been infected with a virus of external origin in the time since its genome was originally sequenced. The phage may have been actively affecting one of the *Lactococcus* cultures. Finally, several unmapped reads showed high identity to members of the genus *Bacillus*. Those reads suggest a low level of *Bacillus* contamination in one of the simulated metagenomes.

**Table 3:** Sequence statistics by library.

	%GC	avg. read length	total reads	Mapped reads	Unmapped reads		
					ambiguous hits to ref orgs	no hits to ref orgs	
						hits NCBI	no hits to NCBI
**Enz+Pyrosequencing**	0.58	202	505962	495428	2354	8004	176
**Enz+Sanger**	0.56	578	11781	9054	364	272	2091
Additional Enz+Sanger	0.59	688	6542	5351	149	267	775
Glycerol Enz+Sanger	0.61	655	6186	4338	294	388	1166
**EnzBB+Sanger**	0.48	563	14418	11552	760	519	1587
Additional EnzBB+Sanger	0.56	665	2040	1258	39	266	477
Glycerol EnzBB+Sanger	0.52	699	1348	718	51	180	399
**DNeasy+Sanger**	0.6	568	14692	11865	759	592	1476
Additional DNeasy+Sanger	0.6	654	2625	1945	90	168	422
Glycerol DNeasy+Sanger	0.59	694	1726	1054	36	155	481

For each library, the average read length, percent G+C, total number of reads, and the numbers of mapped and unmapped reads are given. The unmapped reads fall into 2 categories: 1) those that have BLAST hits to our reference organisms, but cannot be mapped to a single organism because they have high sequence identity to more than one organism or because the sequence identity is below the 95% threshold; and 2) those that do not have BLAST hits to our reference organisms. Reads in the second category are further subdivided into reads that do hit other organisms in the NCBI non-redundant nucleotide database and reads that do not.

### Observed and predicted number of reads for each organism

**Figure 3:**
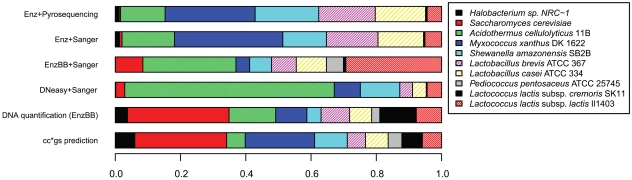
Predicted and observed frequencies of sequence reads from each organism. The fraction of reads assigned to organisms for each sample preparation method is shown at top. The fraction expected given the measured quantities of mixed DNA from each organism assuming unbiased library prep and sequencing is given as “DNA quantification”, and the fraction of reads predicted based on cell count and genome size is given as “cc*gs prediction.” Sampling error was estimated assuming a multinomial distribution (not shown) and indicated that estimates of relative abundance are accurate +/−5% for dominant organisms given the number of Sanger reads obtained, and +/−1% for pyrosequencing reads. Note that the top two bars labeled Enz+Pyrosequencing and Enz+Sanger offer a comparison of Sanger and pyrosequencing technology on the same extracted DNA.

By counting the number of reads mapped to each reference genome and normalizing by the total read count, it is possible to estimate the relative abundance of organisms in each simulated metagenomic sample. Figure 3 shows the frequency at which reads are observed for each organism in our samples. These observed read frequencies can be considered as possibly biased estimates of the organism relative abundance in our simulated environmental samples.

Given that a known quantity of each organism was mixed in the metagenomic simulation, we next investigated whether estimates of organism relative abundance based on sequencing read counts would match the predicted abundance given the way in which our sample was created. To do so, we must first derive a predicted relative DNA abundance based on the known cell count relative abundances. Because we included an equal number of cells per organism in our mixtures, a simple prediction would be that the number of reads per organism in each sequencing library would be directly proportional to their genome sizes. The relative abundance predicted based on genome size and cell counts (cc*gs) is shown in Figure 3. Using the cc*gs predictor of relative organism abundance, we tested whether the observed abundances followed the expected distribution. We found that that cc*gs is a poor predictor of organism abundance in our sequence libraries (χ^2^ test, all p-values <<0.001, Bonferroni multiple test correction). However, some organisms in our experiment such as *Halobacterium* may be polyploid [34], and for many microbes the copy number of the entire (or some segments) of the chromosome can vary depending on growth phase [35], [36] or other factors [37]. Also, the amount of DNA from an organism that is available to become part of a sequencing library depends on the efficiency of the DNA extraction protocol. In a mixed sample, organisms with thick cell walls may yield relatively little DNA, leading to an under-representation of that organism in the final sequencing library [22].

For these reasons, simply counting cells and accounting for genome size may not provide us with an accurate prediction of relative organism DNA abundance. We developed an alternative means to predict the relative DNA abundance of organisms by extracting DNA from a known number of cells of each organism in isolation and quantifying the amount of extracted DNA (see Table 1). We did so using the extraction method (EnzBB) that has been demonstrated in previous studies to achieve the maximum DNA yield from even the most recalcitrant cells [19], [38]. This DNA quantification provides another way to estimate the amount of DNA per cell that we should expect from the simulated metagenomic samples. We predict the reads per organism to be directly proportional to the amount of DNA that can be extracted from each cell. Of course, this prediction based on isolate DNA extraction (DNA quantification) does not provide a perfect expectation of the relative organism abundance in extractions of mixed communities, but it does, at least theoretically, better account for the effects of DNA extraction efficiency and genome copy number per cell. Nevertheless, the observed organism abundance in our sequence libraries does not match the expectation based on DNA quantification (χ^2^ test, all p-values <<0.001.)

While this experiment was not designed to test specific hypotheses about how phylogeny, cell morphology, or genome characteristics may affect the outcome of a metagenomic survey, some interesting observations can be made. For example, because they have been shown to be more recalcitrant to lysis, one might expect that the organisms with the Gram-positive cell wall structure might consistently be under-represented in our libraries relative to the prediction based on isolate DNA extraction. This was not this case in our libraries, where in any given sample, some Gram-positive organisms were more abundant and others less abundant relative to our prediction (Figure 3). One also might expect that closely related organisms that share many genome characteristics would show the same distribution under a given preparation protocol. However, this is not the case with the five lactic acid bacteria, wherein even two strains of the same named species (*Lactococcus lactis*) differ in their read counts by more than an order of magnitude. In the EnzBB library for example, of the 11552 mapped reads, 3389 reads mapped to the *Lactococcus lactis* IL1403 genome while only 86 mapped to the *Lactococcus lactis* SK11 genome (see Table 2 and Figure 3).

The difference in read frequencies among members of the same named species cannot be ascribed to a lack of sequence differences among the two strain's genomes causing a failure in read assignment. Whole-genome alignment using the Mauve genome alignment software [39] reveals the two *Lactococcus* isolates have approximately 87% average nucleotide identity thoughout their genomes and fewer than 1% of subsequences of the length of our reads lack differences to guide taxonomic assignment.

Of course, factors other than DNA extraction efficiency may contribute to differences between the predicted number of reads based on isolate DNA extraction and the observed number of reads. These include 1) cloning bias, which refers to the phenomenon whereby some DNA sequences are more readily propagated in *E. coli* [40]; 2) sequencing bias, which can refer to the propensity of the polymerase enzyme used for Sanger sequencing to stall and fall off when regions of the molecule with secondary structure are encountered [41] or to errors introduced into pyrosequencing reads where there are homopolymeric runs [42]; and 3) computational difficulties with accurately and specifically binning reads. Future studies might attempt to disentangle the contribution of each of these factors to overall bias.

### Comparison of DNA extraction methods

In terms of the relative abundance of organisms based on sequence reads, all metagenomic samples were significantly different from each other and significantly different from the estimated expected distribution (χ^2^ test, p-value <<0.001 for all pairwise comparisons, see Table 2 for data.) *Halobacterium sp*. NRC-1, *Saccharomyces cerevisiae* S288C, and *Lactococcus lactis cremoris* SK11 were under-represented in all libraries relative to the prediction based on isolate DNA extraction, whereas *Acidothermus cellulolyticus* and *Shewanella amazonensis* SB2B were over-represented in every library. Some organisms, *e.g., Pediococcus pentosaceous*, *Lactococcus lactis cremoris* IL1403, and *Myxococcus xanthus* DK 1622 were much more abundant in one library than in others (Figure 3).

The results demonstrate that two libraries created from a single mixture of organisms, but prepared using DNA that has been extracted by different protocols (*i.e.*, Enz, EnzBB, or DNeasy), can produce reads that seem to represent two very different underlying communities. Therefore, the purpose of a metagenomic survey must be taken into consideration when choosing a DNA extraction protocol. While using multiple DNA extraction procedures on a single environmental sample can increase the likelihood that every organism in an environment will be sampled, doing so can also complicate quantitative comparisons of multiple samples.

### Comparison of Sanger vs pyrosequencing

One advantage of sequencing with the pyrosequencing technology over that of clone library-based (Sanger) methods is the elimination of cloning bias. The Enz DNA extraction was split into two samples (Figure 2), one of which was cloned and sequenced using Sanger sequencing while the other was used to construct a library for pyrosequencing. These two libraries, like all others, yielded significantly different taxonomic distributions of reads (all χ^2^ tests have p-value <<0.001.) However, the χ^2^ statistic was lower (χ^2^ = 381.69) than any of the Sanger library pairwise comparisons, all of which had χ^2^>10397. This suggests that the effect of DNA extraction is more pronounced than the bias introduced by clone-based sequencing. Additionally, cloning bias has been shown to be influenced by GC content [20], [39], and in this experiment, the GC content of the Sanger-sequenced sample (56.0% GC) and the pyrosequenced sample (56.7% GC) using the same DNA extraction protocol were very similar. On the other hand, the GC content of the Sanger-sequenced libraries, using different DNA-extraction methods, ranged from 0.48% to 0.61% (Table 3).

The Enz+pyrosequenced metagenome differs from the Enz+Sanger metagenome in the types of reads that failed taxonomic assignment. Whereas very few Enz+Sanger reads failing taxonomic assignment had recognizable sequence identity to organisms in the NCBI non-redundant nucleotide database (547/2638 or 21% of unmapped reads), the majority of the unmapped pyrosequencing reads did have recognizable identity to NCBI database sequences (10171/10347, 98%). Both methods had a modest number of reads that failed taxonomic assignment because the read's sequence identity to the reference organism was below the stringent identity threshold (316 Enz+Sanger reads, between 791 and 2932 Enz+pyrosequencing reads). Additionally, about 0.3% of the Enz+pyrosequencing unmapped reads exhibited sequence identity to an unknown member of the *Bacillus* genus. We speculate that a small amount of *Bacillus* DNA may have entered the Enz+pyrosequencing sample prior to emulsion PCR (see Methods), which may have amplified the contaminant.

### Additional simulated metagenomes

**Figure 4:**
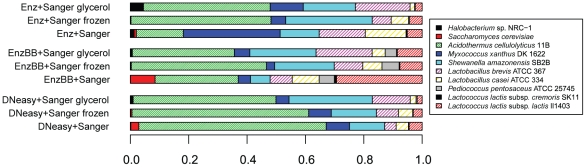
Additional sequence data for three of the simulated metagenomes. Bars represent the observed frequency of organisms in sequenced metagenomes. We constructed and sequenced metagenomes according to the Enz, EnzBB, and DNeasy protocols using the long term frozen isolate culture stocks with glycerol and without glycerol. Reads were mapped to reference genomes as described in Methods. The additional metagenomes show some differences to each of the original libraries. Such differences might be caused by variation across DNA preparations and sequencing runs, age of the frozen samples, or other factors. The libraries constructed using the DNeasy Kit produced the most consistent results.

As mentioned before, the primary purpose of this experiment was to generate sequence data that could be used to test the computational tools that are used to analyze metagenomic sequence data. With this in mind, we opted to use several DNA extraction methods in order to maximize the likelihood of recovering sequence data for every organism in our sample. We did not perform technical replicates for each DNA extraction method. However, *post hoc* comparisons of the different DNA extraction protocols did produce interesting results, prompting us to perform again the same experiments on a smaller scale. While these are not perfect technical replicates, they were performed using exactly the same starting material. These additional simulated metagenomes were created by thawing additional aliquots of the primary frozen culture stocks and mixing them as described below. We did two additional simulations for each of the Enz, EnzBB, and DNeasy protocols and performed Sanger sequencing on the extracted DNA (Figure 4). One of the additional simulations used frozen stock of isolate cultures, the other used frozen stock of isolate cultures with glycerol added to a final concentration of 10%. The so-constructed sequence libraries are not technical replicates of the simulation because they include effects introduced by long-term frozen storage of isolate cultures at −80°C with and without glycerol. Use of glycerol should help prevent cells from lysing, so if large differences were observed between the repeated samples with and without glycerol, it would be reasonable to suspect that cell lysis is an important factor to consider when doing metagenomics with frozen samples.

For each additional simulation, we began by retrieving aliquots of Mix #1 (for the additional EnzBB and DNeasy libraries) or by re-creating Mix #2 (for the Enz library). For the additional libraries using glycerol stocks, both Mix #1 and Mix #2 were re-created from the individual stock cultures. As before, the taxon relative abundance distribution for each library is significantly different from every other library (χ^2^ test, all p-values <0.001). However, if we consider the original libraries to represent an expected organism relative abundance for each DNA extraction protocol, then we can compare the average Chi-square statistic within each DNA extraction protocol to determine which protocol yields the most consistent results. The average Chi-square statistic for the additional libraries is much lower for the DNeasy extraction (average χ^2^ = 377.26) than for either the Enz extraction (average χ^2^ = 5013.12) or the EnzBB extraction (average χ^2^ = 774.96) protocols. This result indicates that the repeatability of the kit extraction method is better than the two other extraction methods (Figure 4). This is in line with expectation, since a possible advantage of kit-based DNA extraction protocols is that variation due to stochastic error should be minimized.

### Conclusion

*In silico* simulations of metagenome sequencing are cheap, quick, and easy, The type of *in vitro* simulation presented here is comparatively expensive, difficult, time-consuming, but captures bias in the metagenomic sampling procedure more faithfully than *in silico* simulations. Studies such as ours add a layer of complexity and biological realism beyond that attainable with computational simulations alone. With *in silico* simulations, one can model complex and highly diverse communities, but the models used to sample reads from isolate genomic data are limited in their ability to capture biases introduced by experimental protocol. In particular, biases in sequence coverage (per genome) can be due to growth conditions, organismal growth phase, DNA extraction efficiency, cloning bias, sequencing efficiency, or relative genome copy number.

In no case did the relative organism abundance in our sequence libraries reflect the known composition of our simulated community. This suggests that sequencing-based methods alone are insufficient to assess the relative abundance of organisms in an environmental sample. If calibrated by another method, such as fluorescent microscopy, sequencing might be more useful in this regard. The results also highlight the need to standardize as many laboratory techniques as possible when comparing metagenomic samples across environments, timescales, or environmental conditions. Currently, there is no standard approach for metagenomic surveys, making it difficult to make useful inferences when comparing data among different studies.

It is important to note that the purpose of a given metagenomic sampling effort will vary, and the methods used should be chosen to best suit that purpose. For example, here we found that using a kit-based DNA extraction protocol produced the most consistent results with repeated sampling. This is important if the goal of a study is to track differences across environments, treatments, or timescales. However, if the goal is to fully catalog all organisms or to know with certainty the relative abundance of organisms in a sample, our results suggest that the kit-based DNA extraction could offer the worst performance of the methods tested here. Of course, there are other factors to consider: the DNA yield from kit-based DNA extractions is considerably lower than alternative methods, it is typically of a lower molecular weight, and it is more costly to acquire.

Our ability to make strong conclusions about the source of variation across samples is unfortunately limited by our lack of technical replicates. However, we find the magnitude of this variation striking, even in this simple, well-understood, artificially constructed microbial “community.” Future experiments to tease apart the sources of bias, especially those designed with specific natural communities in mind, will be valuable. In addition to providing sequence data that can be used for benchmarking analytical techniques for metagenomics, it is our hope that this type of simulation can help aid model development for future *in silico* simulations. For this purpose, sequence data generated in our study is available via the IMG/M [43], on the BioTorrents [44]file sharing site (http://www.biotorrents.net/details.php?id=47), and via the NCBI's Trace and Short Read Archives.

## Methods

### Laboratory Methods

#### Cell culture

*Myxococcus xanthus* DK1622 cells were grown in CTTYE (1% Casitone [Difco], 10 mM Tris-HCl (pH 7.6), 1 mM KH_2_PO_4_, 8 mM MgSO_4_) broth at 33°C with vigorous aeration. Cells were harvested when a Klett-Summerson colorimeter read 100 Klett units, or approximately 2×10^8^ cells/ml. *Acidothermus cellulolyticus* 11B was grown in liquid culture at 55 degrees C on a shaker at 150 rpm. The growth medium consisted of American Type Culture Collection medium 1473, modified by use of glucose (5 g/l) in place of cellulose, pH 5.2–5.5. The five lactic acid bacteria were provided as streaked MRS agar plates, from which single colonies were used to start pure cultures in liquid MRS broth. *Halobacterium sp*. NRC-1 (ATCC#700922), *Saccharomyces cerevisiae* S288C (ATCC#204508), and *Shewanella amazonensis* SB2B (ATCC# BAA-1098) were obtained as freeze-dried stocks and used per recommended protocol to start cultures in the prescribed media. Cultures were grown 12–48 hours until turbid. The cell density of each culture was determined by counting DAPI-stained cells using a Cytopeia Influx flow cytometer. Immediately after counting, the cultures were aliquoted into ten 2 mL cryotubes, flash-frozen in liquid nitrogen and stored at −80°C. Glycerol was added to one of the tubes before freezing to make a 10% glycerol stock solution (except for the *Myxococcus xanthus*, which was provided as flash-frozen liquid culture.)

#### Mixing

Two techniques were employed for mixing. **Mix #1:** One tube of each of the ten cultures was thawed on ice. An aliquot from every tube was added to a single new tube such that each organism contributed an equal number of cells to the final mixture. This final mixture was aliquoted into four 2 mL cryotubes which were flash-frozen and returned to −80°C. Immediately prior to DNA extraction, one of the 2 mL cryotubes of the final mixture was centrifuged for 10 minutes at 10,000 rpm to pellet cells. The supernatant was removed, and the cell pellet was resuspended in TES buffer (10 mM Tris-HCl pH 7.5, 1 mM EDTA, 100 mM NaCl). **Mix #2:** One tube of each of the ten cultures was thawed on ice. An aliquot from every tube was transferred to a new tube so that the new set of tubes contained an equal number of cells per tube. Immediately prior to DNA extraction, each tube was centrifuged for 10 minutes at 10,000 rpm to pellet the cells. Each cell pellet was resuspended in the lysis buffer that is provided with the DNeasy kit (Qiagen, Valencia, CA), and the contents of all ten tubes were pooled into a single tube.

#### DNA extraction

DNA Prep #1 (**EnzBB**): The resuspended cells were incubated with a final concentration of 50 U/uL lysozyme (Ready-Lyse, Epicentre Technologies) at room temperature for 30 minutes. Further lysis was accomplished by the addition of proteinase-k and SDS to a final concentration of 0.5 mg/mL and 1%, respectively, and incubation at 55°C for 4 hours. Finally, the lysate was subjected to mechanical disruption with a bead beater (BioSpec Products, Inc., Bartlesville, OK), on the Homogenize setting for 3 minutes. Protein removal was accomplished by extracting twice with an equal volume of 25∶24∶1 phenol:chloroform:isoamyl alchol. The aqueous phase was incubated at −20°C for 30 minutes with 2.5 volumes of 100% ethanol and 0.1 volumes of 3 M sodium acetate before centrifugation at 16,000 g for 30 minutes at 4°C. The DNA pellet was washed with cold 70% ethanol and allowed to air dry before resuspension in TE (10 mM Tris-HCl pH 7.5, 1 mM EDTA.) DNA quantitation was performed using the Qbit fluorometer (Invitrogen).

DNA Prep#2 (**DNeasy**): Qiagen's DNeasy kit (Qiagen, Valencia, CA) per manufacturer's protocol for bacterial cultures.

DNA Prep #3 (**Enz**): Identical protocol to DNA Prep#1 but without the bead beating step.

#### Library construction and sequencing

Three small-insert (∼2 kb) libraries were constructed by randomly shearing 10 µg of metagenomic DNA using a HydroShear (GeneMachines, San Carlos, CA). The sheared DNA was electrophoresed on an agarose gel, and fragments in the 2–3 kb range were excised and purified using the QIAquick Gel Extraction Kit (Qiagen, Valencia, CA). The ends of the DNA fragments were made blunt by incubation, in the presence of dNTPs, with T4 DNA Polymerase and Klenow fragment. Fragments were ligated into the pUC18 vector using the Fast-Link(TM) Ligation Kit (Epicentre, Madison, WI) and transformed via electroporation into ElectroMAX DH10B(TM) Cells (Invitrogen, Carlsbad, CA) and plated onto agar plates with X-gal and 150 mg/mL Carbenicillin. Colony PCR (20 colonies) was used to verify a >10% insertless rate and ∼1.5 kb insert size. White colonies were arrayed into 384-well plates for sequencing.

For Sanger sequencing, plasmids were amplified by rolling circle amplification using the TempliPhi(TM) DNA Sequencing Amplification Kit (Amersham Biosciences, Piscataway, NJ) and sequenced using the M13 (−28 or −40) primers with the BigDye kit (Applied Biosystems, Foster City, CA). Sequencing reactions were purified using magnetic beads and run on an ABI PRISM 3730 (Applied Biosystems) sequencing machine.

The library for pyrosequencing was constructed using ∼5 mg of metagenomic DNA, which was nebulized (sheared into small fragments) with nitrogen and purified with the MinElute PCR Purification Kit (Qiagen, Valencia, CA). The GS20 Library Prep Kit was used per manufacturer's protocol to make a ssDNA library suitable for amplification using the GS20 emPCR Kit and then prepared for sequencing on the Genome Sequencer 20 Instrument using the GS 20 Sequencing Kit.

#### Sequence data submission

All Sanger-generated sequence data have been submitted to the NCBI Trace Archives, with Trace Archive ID numbers 2261924487 through 2262015859. The pyrosequencing-generated sequence data have been submitted to the NCBI Short Read Archives with Accession number SRA010765.1.

### Sequence Analysis

#### Sequence trimming

Vector sequences were removed with cross_match, a component of the Phrap software package [45] and low-quality bases, *i.e.* those with a PHRED [46] quality score of Q> = 15, were converted to “N”s using JAZZ, the JGI's in-house genome sequence assembly algorithm.

#### Taxonomic assignment of reads

We mapped reads back to reference genomes by means of BLAST search [33]. A BLAST database containing the nucleotide sequence of each of the ten genomes (chromosomes and plasmids) was constructed. Reads were searched against that BLAST database, and low-scoring hits (e-value>0.0001) were discarded except for the pyrosequencing-generated reads, for which a threshold of 0.01 was used. Reads not passing BLAST's low complexity filter were considered to have failed QC, this happened frequently for reads containing a large number of <Q15 bases replaced with N. Some reads contained a high fraction of N bases but still passed the low complexity filter, such reads frequently had no significant hit to the 10 reference organisms. Reads with hits were assigned to the genome corresponding to their top BLAST hit only if the top hit had sequence identity >95% and the next highest hit to a different organism had a bit score at least 20 points lower. Such reads are considered “mapped.” In order to investigate possible contamination in sequence libraries, reads without hits were searched against the NCBI non-redundant amino acid database in parallel using mpiBLAST [47].
